# Chloralism

**Published:** 1875-10

**Authors:** 


### Chloralism.

*The Chemist and Druggist* quotes from *Belgravia* for April, extracts from "The Confessions of an English Chloral Eater, written by Dr. Gordon Stables, of the Royal Navy. The narrative is not apparently intended for fiction, though evidently written with that tint of extravagance which was requisite to make it a popular magazine article. The following passages are quoted from the history, as well calculated to check the too easy reliance on the innocence of the medicine which early reports of its action were calculated to engender:

"The stimulation," says the doctor, "is not like that caused by opium or alcohol: it is not exhilarating, and does not incite to action either mentally or bodily. But the subject of the influence rises for a time above all his cares, or sorrows, or fatigue, and seems to look on life through the medium of a rose-tinted glass. But while care and sorrow are forgotten, and a strange dreamy sense of perfect ease, comfort, and happiness takes their place, all affection and love are likewise banished. He is apathetic, and cares for nothing save his own sense of comfort. He is, if I might so express it, merely a living, breathing vegetable. In this state the confirmed chloral eater would stand by the death-bed of his nearest and dearest a passive spectator, if not indeed actually smiling; and for the same reason he would stand quietly on the scaffold until executed. If the dose is repeated without the chloralist lying down, speech becomes indistinct, the eyelids drop, and the gait in walking is affected, just as in drunkenness from alcohol. The chloralist drunk in the first degree, is by no means an unpleasant companion. A stranger could mark nothing unusual about him; he is genial, and although rather languid and by no means bright in conversation, he is at all events a good listener, and is easily pleased, although his smiles often partake of the simpering or hysterical order; and, too, he is at times easily roused into an outbreak of furious passion, which dies away just as suddenly as it came, leaving no trace behind. But of course every one will not be effected precisely alike, as much depends on the idiosyncrasy or innate peculiarities of the chloralist."

The writer's personal experience with chloral commenced in December, 1871. He was suffering from sleeplessness, and, tempted by the medical praises of the new hypnotic, he began with doses of 20 grains. The "sweet restorer" answered promptly this enchanted call, and the patient, delighted with the power with which he found himself possessed, continued to use it. Very strikingly he described the nature of the sleep which he could thus produce. "I observed," he says, "that on awakening in the morning I felt as if actually no space of time had intervened since I lay down. My life seemed a continuous never-ending day; I had no satisfaction from my sleep, and felt dispirited in consequence. If I had only taken warning now! But I did not; for this same peevishness is the earliest symptom of that coming irritability, or chronic congestion of the brain, which the continuous use of chloral never fails to produce. About two months after I had begun taking chloral, I first became sensible of a strange heat on the top of my head, together with a sense of fulness in the head. My nerves, too, began to be shaken. I could do things slowly, but any hurry or excitement at once confused me."

All this time, he says, he had no suspicion that it was the hydrate of chloral that was doing the mischief, and was treating himself for brain congestion. Things grew rapidly worse, till at the end of June, 1872, he writes:

"My bodily sufferings are very great, and my mind is a mere chaos. My face is *so* thin and white and worn, that I start at my own image in the glass. My eyes are constantly dilated, and the least excitement runs my pulse from sixty to a hundred. Towards evening my head feels as if frozen, and I sit in a benumbed stupor until bed-time. Undressing I feel as one of the labors of Hercules, and has to be done by degrees. I do not take my chloral—three drachms, enough to kill as many men—until I am in bed, and the house is perfectly still; for the slightest noise would necessitate a double dose. When all is quiet, I drink and

. . . . . . . . . . . . . .

"If I were not writing these confessions as a warning to others, I would draw a veil over my last experiences of chloral hydrate. For the first fortnight in December never less than five drachms of this medicine was my nightly dose. From the time I arose in the morning—for I still left bed daily, having a horror of it—my sufferings were extreme. I had now lost all power of reading, writing, or speaking aloud; any attempt to do either was excruciating brain agony, and if persevered in, fainting followed. I could hardly move my head from the pillow or sit erect, while my eyes seemed starting from their sockets if I attempted to walk. But towards night—well, if all of mental, all of bodily suffering I ever endured in life could be compressed into one hour, it would not exceed the torments I then underwent. Every vein in my body seemed swollen to double the size and inflamed along the whole length, while the restlessness was so distressing that I could not lie for five minutes in any one position. Add to this that time seemed indefinitely long—minutes as hours and hours as days—and you will have some faint notion of my experience of the 'grand new remedy for sleeplessness that had no after-effects.'"

From the time of totally abandoning the use of the poison, however, he began to mend, though his recovery, he tells us, was a long and tedious one. Doubtless Dr. Stables' object is to warn his readers against indulging in chloral at their own discretion, not absolutely to condemn it when administered under medical direction.—*New Remedies.*

